# Association between gut microbiota and psychiatric disorders: a systematic review

**DOI:** 10.3389/fpsyg.2023.1215674

**Published:** 2023-08-03

**Authors:** Carmen Grau-Del Valle, Javier Fernández, Eva Solá, Inmaculada Montoya-Castilla, Carlos Morillas, Celia Bañuls

**Affiliations:** ^1^Department of Endocrinology and Nutrition, University Hospital Doctor Peset, Foundation for the Promotion of Health and Biomedical Research in the Valencian Region (FISABIO), Valencia, Spain; ^2^Department of Medicine, University of Valencia, Valencia, Spain; ^3^Department of Personality, Assessment and Psychological Treatment, University of Valencia, Valencia, Spain

**Keywords:** gut microbiota, psychiatric disorders, human, dysbiosis, gut-brain axis

## Abstract

**Introduction:**

In recent years, it has been described that the dysbiosis of the intestinal microbiota plays a transcendental role in several pathologies. In this sense, the importance of the gut microbiota in the gut-brain axis, with a bidirectional communication, has been demonstrated. Furthermore, the gut microbiota has been linked with mood disorders and neuropsychiatric disorders.

**Methods:**

A systematic review of two databases – PubMed and Scopus – was carried out following PRISMA guidelines. We included original studies in humans with a control group published in the last 11 years, which were assessed by the Critical Appraisal Skills Program (CASP) to confirm their quality. Eighteen articles met all the selection criteria.

**Results:**

A review of the articles revealed an association between psychiatric disorders and different bacterial phyla. The studies we have reviewed have demonstrated differences between subjects with psychiatric disorders and controls and highlight a clear relationship between depression, stress, autism spectrum disorder (ASD), psychotic episodes, eating disorders, anxiety and brain function and the gut microbiota composition.

**Conclusion:**

A reduction of fermentative taxa has been observed in different psychiatric disorders, resulting in a decrease in the production of short-chain fatty acids (SCFAs) and an increase in pro-inflammatory taxa, both of which may be consequences of the exacerbation of these pathologies.

## 1. Introduction

The gut microbiota is a complex ecosystem consisting of bacteria, viruses, fungi and archaea, though the bacterial kingdom is the most studied of these populations (Thursby and Juge, 2017).

Several studies have linked the role of the gut microbiota to overall health status (Cresci and Bawden, 2015). In this way, some bacterial taxa have emerged as important treatments of dysbiosis/unbalanced microbiota (Cristofori et al., 2021). In addition, the inclusion of probiotics and prebiotics in the diet and fecal microbiota transplantation are validated strategies in the treatment of specific infections, especially those caused by resistant strains of *Clostridioides difficile* (Mills et al., 2018; Sandhu and Chopra, 2021). Moreover, strategies based on modulation of the gut microbiota appear as promising options to treat a wide variety of other pathologies, such as intestinal bowel disease, inflammatory diseases, metabolic diseases, cancer, and other pathologies (Marchesi et al., 2016; Yao et al., 2021; Fernández et al., 2022). However, further research is needed to verify the suitability of their clinical use in these diseases.

Emotional states are processes that regulate brain and body, and represent a reciprocal brain–body dialogue (Colombetti and Zavala, 2019). In recent years, the interaction between the gut microbiota and the central nervous system has acquired a special significance referred to as the ‘gut-brain axis’ (Du et al., 2020), which is a two-way communication between gut bacteria and the brain that takes place via the nervous, endocrine and immune systems. This bidirectional communication involves neuronal modulation, immune response and hormone release (Neufeld et al., 2011; Selkrig et al., 2014). As it is a bidirectional response, it involves regulation of the permeability of the intestinal epithelium and the blood–brain barrier. The response of intestinal microbiota is mediated by various metabolism products, including short-chain fatty acids (SCFAs); bacterial neurotransmitters such as gamma-aminobutyric acid (GABA) or serotonin; modulators such as quinolinic acid, which modifies the immune system; and hormones such as cortisol (Fernández et al., 2016; Puricelli et al., 2021; Chen et al., 2022). The microbiome is also thought to influence brain function, behavior (Vuong and Hsiao, 2017) and mental health (Valles-Colomer et al., 2019).

Alterations in the human gut microbiota composition have been linked with mood disorders and neuropsychiatric disorders (Huang et al., 2019) and, in turn, with neurotransmitters imbalances (Frankiensztajn et al., 2020). Increasing evidence has linked the gut microbiota with symptoms of autism spectrum disorder (ASD) that are regularly affected by gastrointestinal problems and the gut microbiota dysbiosis (Yang et al., 2018). Furthermore, many patients who experience gastrointestinal discomfort are more likely to present mental disorder comorbidities (Huang et al., 2019). In this sense, an alteration of the gut microbiota brain axis has been associated with autistic behaviors (Li and Zhou, 2016). In terms of emotional states, this axis is also involved in the regulation of stress-related responses (Foster et al., 2017), specifically the hypothalamus-pituitary-adrenocortical (HPA) axis. The gut microbiota is closely connected to the development and function of the HPA axis (Frankiensztajn et al., 2020). In this regard, alterations in the metabolic, immune and endocrine systems have been found in patients with depression, pointing to an association between the pathophysiology of depression and the gut microbiota (Caspani et al., 2019). In addition, the gut microbiota of depressed patients are significantly different from those of healthy controls (Liang et al., 2018). Thus, modulation of the gut microbiota and neuroinflammation could alter brain function and have an influence on depressive and anxiety-like behaviors (Koopman and El Aidy, 2017). With respect to severe mental illness, dysbiosis has also been identified as a comorbidity of schizophrenia (Castro-Nallar et al., 2015) and has been associated with the severity of psychotic symptoms and global functioning in patients during their first episode of psychosis (Nguyen et al., 2018). The interaction with the gut-brain axis has also been studied in the context of eating disorders (Seitz et al., 2020). In this sense, some research suggests that the gut microbiota is altered in patients with anorexia nervosa (Hanachi et al., 2019), since it seems to play a role in different metabolic functions (regulation of weight gain, insulin secretion and energy production from food) (Tremaroli and Bäckhed, 2012). The gut microbiota has also been linked to neurodegenerative diseases and may play a key role in the aging process, the loss of quality and strength of muscle, the loss of skeletal mass and in the decline in cognitive function (Ni et al., 2019). The aim of the present review is to provide an overview of the results of human clinical studies published over the last 11 years that highlight the relationship between gut microbiota and psychiatric disorders.

## 2. Methods

Based on the Preferred Reporting Items for Systematic Reviews and Meta-Analysis (PRISMA) guide (Hutton et al., 2016), we employed an evidence-based model to frame a PICO question model (PICO: Participants, Intervention, Control, and Outcomes).

The following question was posed: “Is there an association between psychiatric disorders and gut microbiota?” Participants: individuals diagnosed with psychiatric disorders. Interventions: untreated or treated (dietary intervention, psychological programs, antipsychotic drugs, probiotics or microbiota transfer therapy…) individuals. Controls: healthy individuals, of similar age and weight, with no psychiatric disorders. Outcome measures: microbiota content and diversity in individuals with psychiatric disorders.

### 2.1. Selection of articles

Searches of the databases PubMed (National Library of Medicine Washington, DC, United States) and Scopus (Elsevier B.V) were conducted up to January 2023, using the following keywords: “Microbiota,” “Psychological disorders” and “Human.” The criteria for inclusion of articles were: original articles, clinical trials or randomized controlled trials carried out in humans and providing reports on key aspects of mood and mental state, particularly psychiatric disorders, carried out in the previous 11 years (2012–2023), in which a control group had been included.

The following exclusion criteria were established: review articles, systematic reviews, letters to the editor and meta-analyses.

**Figure 1:**
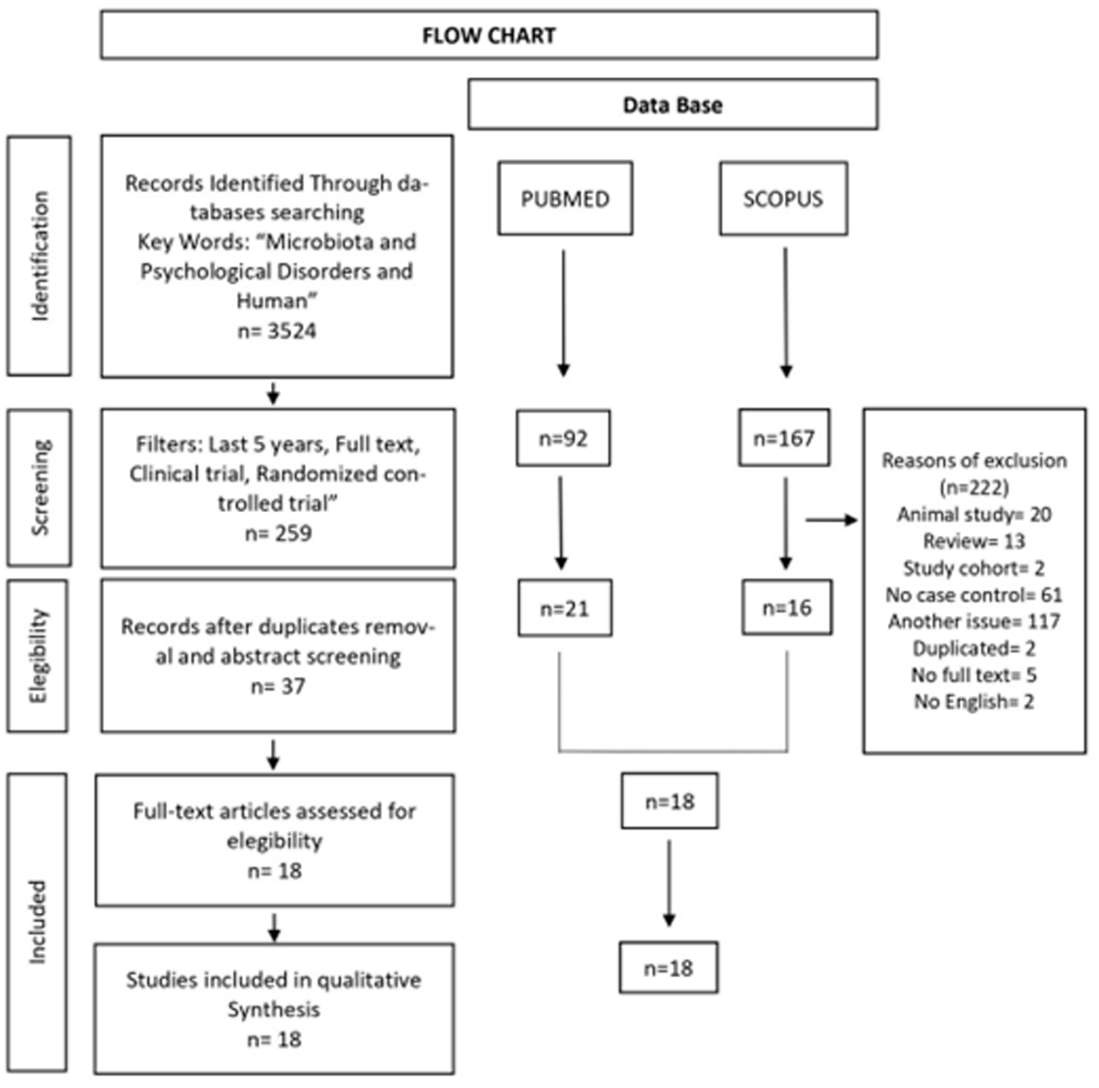
Flow chart of the systematic review according to PRISMA guidelines.

Once the articles were identified, they were screened reading initially the titles and abstracts. The next step involved an examination of the full text based on the established eligibility criteria. Articles whose content did not involve microbiota and their relationship with psychiatric disorders were excluded. Duplicated articles were also eliminated. Thus, only that met the criteria of the PICO question remained (Figure 1).

### 2.2. Quality of articles

To perform a critical reading of the studies that met all the selection criteria, the Critical Appraisal Skills Program (CASP) was used (Zeng et al., 2015). The CASP is organized in the following three sections: (A) Are the results of the study valid? (B) What are the results? (C) Can these results help us in our environment/area? Each question that could be answered affirmatively contributed one point to the quality score allotted to an article. The checklist had a maximum score of 11 points. In case–control studies, the scores represent the following items: Item 1: Study issue is clearly focused; Item 2: Cohort is recruited in an acceptable way; Item 3: Exposure is accurately measured; Item 4: Outcome is accurately measured; Item 5: Confounding factors are addressed; Item 6: Follow-up is long and complete; Item 7: Results are clear; Item 8: Results are precise; Item 9: Results are credible; Item 10: Results can be applied to the local population; and Item 11: Results fit with available evidence. In randomized clinical trials, the scores represent the following items: Item 1: Was the study issue is clearly focused?; Item 2: Was the assignment of treatments to patients randomized?; Item 3: Were all the patients who entered the trial properly accounted for at its conclusion?; Item 4: Were patients, health workers and study personnel “blind” to the treatment?; Item 5: Were the groups similar at the start of the trial?; Item 6: Aside from the experimental intervention, were the groups treated equally?; Item 7: How large was the treatment effect?; Item 8: How precise was the estimate of the treatment effect?; Item 9: Can the results be applied to the local population, or to your context?; Item 10: Were all clinically important outcomes considered?; Item 11: Are the benefits worth the harms and the costs?

### 2.3. Dimensions of psychiatric symptoms

We reviewed the following psychiatric disorders; Depression, Autism Spectrum Disorders (ASD), Attention Deficit Hyperactivity Disorder (ADHD), Stress, Cognitive Decline, Binge Eating Disorder, Anorexia Nervosa, Anxiety, Posttraumatic Stress Disorder (PTSD) and Psychotic Episode.

Psychiatric l symptoms were measured with a variety of validated self-report scales and the Diagnostic and Statistical Manual of Mental Disorders (DSM-5). The depressive disorder in question was evaluated with 20 items of the Center for Epidemiologic Studies-Depression Scale (CES-D) (Radloff, 1977), designed to measure self-reported symptoms associated with depression experienced in the past week, and by means of different questions that evaluate well-being and self-rated health such as: Hamilton Depression Scale (HAMD-24 items) (Hamilton, 1960); Self-rating Depression Scale (SDS), with 20 items used to measure the level of depression (Holmstrøm et al., 2004); and Hamilton Depression Scale (HAMD-17), a depression scale with 17 items (Hamilton, 1960).

Diagnosis of ASD was performed using the Autism Diagnostic Interview-Revised (ADI-R) (Lord et al., 1994). The Autism Diagnostic Interview (ADI) is composed of 35 items in 4 main areas: social/reciprocal interaction, communication, speech and language, and restricted/repetitive behavior (Le Couteur et al., 1989). Other mechanisms used to measure autism were the Patient Global Impressions-III (PGI-III) scale (Adams et al., 2011) and the Childhood Autism Rating Scale (CARS) (Shaffer, 1983). The Aberrant Behavior Checklist (ABC) assesses problem behaviors, the Social Responsiveness Scale (SRS) is a 65-item scale that assesses social impairments, and the Vineland Adaptive Behavior Scale II (VABS-II) (Kang et al., 2017) measures functioning level in four different domains: communication, daily living skills, socialization, and motor skills. The Chinese Classification of Mental Disorders (CCMD-3) (Wang et al., 2020) consists of four subscales: speech/language/communication (14 items), sociability (20 items), sensory/cognitive awareness (18 items) and health/physical/behavior (25 items). Severity of autism was assessed with the Autism Treatment Evaluation Checklist (ATEC) (Mahapatra et al., 2018). Finally, the Children’s Global Assessment Scale (CGAS) (Shaffer, 1983), with a score between 1 and 100, assesses aspects related to a child’s psychological and social functioning, and the ADHD Rating Scale IV (ADHD-RS-IV) (Zhang et al., 2005) contains 18 items directly linked to DSM-IV diagnostic criteria for ADHD.

Regarding measures of stress levels, the following instruments were used: the SISCO inventory of academic stress (Castillo et al., 2018; Manrique-Millones et al., 2019), which measures the adverse effect of stress on behavior and health; the Generalized Anxiety Disorder-7 (GAD-7) (Spitzer et al., 2006), consisting of 7 items that measure symptoms of worry and anxiety; the Patient Health Questionnaire-9 (PHQ-9) (Kroenke et al., 2001), a 9-item self-report measure used to assess severity of depression; CASP-5, a structured diagnostic interview used to diagnose PTSD based on DSM-5 criteria (Feng et al., 2012; Bovin et al., 2016); and the Childhood Trauma Questionnaire (CTQ) (Berg, 1988), which consists of 28 self-report items used to calculate a total childhood trauma score by adding the scores obtained on 5 trauma subscales.

The Mini-Mental State Examination (MMSE) (Feng et al., 2012) is a measure of *cognitive function*, and the Clinical Dementia Rating (CDR) (Hughes et al., 1982; Berg, 1988; Morris, 1993) determines signs of cognitive impairment.

For eating disorders, the 50-item Eating Disorder Diagnosis Questionnaire (Q-EDD) (Mintz et al., 1997) was used. The short version of the Dutch Eating Behavior Questionnaire (DEBQ) (Bailly et al., 2012) was used to assess restrained, emotional, and external eating behavior, and the Positive and Negative Affect Schedule (PANAS) (Watson et al., 1988; Brasseur et al., 2013) was employed to measure general mood and emotion regulation abilities.

The severity of eating and psychopathology disorders was assessed with the Eating Disorder Inventory-2 (EDI-2) (Garner et al., 1983), a questionnaire that explores typical cognitive and behavioral characteristics of eating disorders, with a total of 91 items and 11 subscales. Anorexia nervosa was diagnosed with a structured interview according to the Diagnostic and Statistical Manual of Mental Disorders-IV-TR criteria (Morita et al., 2015). The Symptoms Checklist-90-Revised (SCL90) (Derogatis and Savitz, 1999) with 90 items and 9 dimensions, assesses general psychopathology. The State Trait Anxiety Inventory (STAI) (Steer et al., 1999) is composed of 20 items and 2 subscales (STAI-1/STAI-2) and evaluates current anxiety status. The Beck Depression Inventory- II (BDI-II) (Beck et al., 1996; Steer et al., 1999) was used as a self-report measure of major depressive disorders, with 21 items divided into 2 subscales. The severity of psychotic symptoms was assessed by the Brief Psychiatric Rating Scale-Extended (BPRS-E) (Garner et al., 1983), which has three domains (alogia, anhedonia-asociality and avolition-apathy), the Scale for the Assessment of Negative Symptoms (SANS) (Andreasen et al., 2010) and the Global Assessment of Functioning (GAF) scale (Schwarz et al., 2018).

The generalized anxiety disorder was evaluated using the Hamilton Anxiety Scale (HAMA-14), which includes 14 items on a 5 point scale (Hamilton, 1959); the Five Factors Inventory-Neuroticism Subscale (FFI-N), that involves 12 items in the questionnaire and is rated on a 5 points scale (Costa and McCrae, 1995); the Chinese version of Illness Perception Questionnaire Revised (IPQ-R), which consists of 38 items and seven dimensions of the disease course (Weinman et al., 1996); the Twenty- Item Toronto Alexithymia Scale (TAS-20), that includes 20 items and three dimensions (difficulty describing feeling, difficulty identifying feeling and externally oriented thinking) (Bagby et al., 1994); the Patient Health Questionnaire- 15 (PHQ-15) which consists of the 15 most common somatic symptoms and is used to assess the severity of somatic symptoms (Kroenke et al., 2001); the Cognitive Emotion Regulation Questionnaire-Chinese Version (CERQ), whose scale contains 36 items and is used to assess the cognitive strategies used by individuals in coping with negative events (Zhu et al., 2008); the Connor-Davinson Resilience Scale, which consists of 25 items and was employed to assess ability to endure stress or pain and to cope with adversity (Bangsgaard Bendtsen et al., 2012); and the Mindful Attention Awareness Scale, composed of 15 items and used to measure the capacity for sustained attention to and awareness of the present moment experience (Dunstan et al., 2017).

## 3. Results

Three thousand five hundred twenty-four articles were identified with the keywords (microbiota, psychological disorders and human) and the following filters were applied to both data bases (yielding 92 articles in PubMed and 167 articles in Scopus): last 11 years (2012–2022); full text; clinical trial; randomized controlled trial. We removed 20 articles about animal studies, 13 systematic reviews, 2 cohort study articles, 61 no case–control articles, 117 articles dealing with a topic other than ours, 5 articles without full text, 2 duplicated articles and 2 articles not written in English, thus rendering a new total of 18 articles. Of these, 15 papers were case–control studies and 3 were randomized clinical trials (Figure 1). Table 1 shows the general characteristics and results of each of the 18 articles. The CASP quality assessment of the reviewed case–control studies and randomized clinical trials is shown in Tables 2, 3, respectively. The total quality score of case–control studies reached a maximum of 9/11 and that of the randomized clinical trial studies reached a maximum of 8–10/11.

**Table 1:** Characteristics of the studies included in the systematic review.

Author, Year	Study design	Study groups (N)	Mean Age (years)	Methods diagnostic	Psychiatric disorders	Gut microbiota	Intervention/follow-up time	Association between Gut microbiota and psychiatric disorders
Uemura et al. (2019)	Randomized controlled trial	IG = 22 CG = 22	62.0 63.3	Center for epidemiologic studies depression scale (CES-D). Subjective well-being and self- rated health with questions.	Depressive symptoms Subjective well-being Self-rated health	*Bacteroidota* − IG *Lactobacillales* + IG *Bacteroidaceae* − IG *Streptococcus* − IG *S. thermophilus* + IG *Bifidobacterium* + IG *Veillonella parvula* + IG	Dietary intake. Referring to the Japanese food guide spinning top/ 8 Weeks.	*Streptococcus thermophilus* contributed to the improvement of the obesity and depressive symptoms. Increased *Bifidobacterium bifidum* and *Veillonella parvula* species contributed to improve physical and psychological indicators.
Chen et al. (2021)	Case Control	IG = 62 CG = 46	39.5 36.0.9	Hamilton Depression Scale (HAMD-17) and e Diagnostic and Statistical Manual of Mental Disorders, 5th Edition’s (DSM-5).	Major Depressive Disorder (MDD)	*Bacteroidota* + IG *Pseudomonadota* + IG *Fusobacteria* + IG *Enterobacteriaceae* + IG *Tannerellaceae* + IG *Burkholderiaaceae* + IG *Campylobacteraceae* + IG *Corynebacteriaceae* + IG *Clostridia* + IG	Untreated	HAMD scores were positively correlated with levels of *Anaerotruncus, Parabacteroides,* and *Anaeroglobus.*
Kang et al. (2017)	Case- Control	IG = 18 CG = 20	10.8 11.4	Interview ADI-R. General physical health examination. Gastrointestinal Symptom Rating Scale (GSRS) The daily stool records (DSR) The Parent Global Impressions-III (PGI-III) The Aberrant Behavior Checklist (ABC) The Social Responsiveness Scale (SRS) The Vineland Adaptive Behavior Scale II (VABS-II)	Autism Spectrum Disorders (ASD)	*Bifidobacterium* − IG *Prevotella* − IG *Desulfovibrio* − IG	Microbiota Transfer Therapy (MTT): oral vancomycin, MoviPrep, SHGM y Prilosec./18 Weeks	At the end of MTT, bacterial diversity was increased in children with ASD. *Bifidobaterium, Prevotella* and *Desulfovibrio* were increased after MTT in ASD.
Stevens et al. (2019)	Randomized Control Trial	IG = 10 CG = 7	10.2 9.3	The Children’s Global Assessment Scale (CGAS) The ADHD Rating Scale IV (ADHD-RS-IV)	Attention-deficit/hyperactivity disorder (ADHD)	*Bifidobacteriales* − IG *Actinomycetota* − IG − CG *Pseudomonadota* + IG *Bacteroidota* + IG + CG *Bacillota* − IG − CG *Collinsella aerofaciens* + IG	Capsules micronutrient/10 Weeks	An increase in *Actinomycetota* was associated with ADHD –IV − RS. After treatment, a low abundance of *Bifidobacterium* was associated with a low ADHD-IV-RS score.
Márquez-Morales et al. (2021)	Case–Control	IG = 27 CG = 18	20.0–25.0	The SISCO Inventory of Academic Stress.	The academic stress	*Bacteroidota* + IG *Bacillota* + IG + CG *Gammapseudomonadota* (No differences)	The fermented beverage with lactic acid bacteria (FBLAB).	Consumption of fermented beverage significantly increased the phyla *Bacillota* and *Bacteroidota* and were associated with a reduction in stress-related symptoms
Khine et al. (2020)	Randomized Control Trial	IG = 46 CG = 77	65.0 67.0	The neuropsychological diagnosis A food frequency questionnaire (FFQ) Mini-Mental State Examination (MMSE) Clinical Dementia Rating (CDR) and battery of standard neuropsychological tests	Cognitive decline	*Ruminococcus* + IG *Ruminococcaceae* + IG *Coprococcus* + IG *Parabacteroides* + IG *Enterobacteriaceae* − IG *Fusobacterium* − IG *Phascolarctobacterium* − IG	Mindful Awareness Program (MAP) 9 months	*Ruminococcaceae* was related with Digit Span Backward; *Coprococcus* was related with Color Trails Test 2, Digit Span Backward and Block Design. *Parabacteroides* was related with Digit Span Backward and Semantic Fluency Span. *Enterobacteriaceae* was negatively associated with Block Design and Semantic Fluency Span. *Fusobacterium* was negatively correlated with Digit Span Backward and Color Trails Test 2; and *Phascolarctobacterium* was negatively associated with Memory Domain.
Xu et al. (2017)	Case control	IG = 5 CG = 5	31.8 32.0	Generalized Anxiety Disorder-7 (GAD-7) Patient Health Questionnaire-9 (PHQ-9)	Stress Chronic	*Prevotella* + IG *Paraprevotella* − IG *Odoribacter* − IG *Veillonella* − IG *Ruminococcus* − IG	Untreated	The prevalence of *Prevotella* was higher in chronic stressed patients. Lower concentrations of *Paraprevotella*, *Odoribacter*, *Veillonella* and *Ruminococcus* were showed in chronic stressed patients.
Leyrolle et al. (2021)	Case control	IG = 42 CG = 59	18.0–65.0	Eating Disorder Diagnostic (Q-EDD). Semi-structured interview conducted. Emotion Regulation abilities (PANAS). Dutch Eating Behavior Questionnaire (DEBQ). Profile of Emotional Competence (PEC). The Scale of positive and negative experience (SPANE).	Binge Eating Disorder	*Anaerostipes* + IG *Roseburia* + IG *Bilophila* + IG *Bifidobacterium* + IG *Sutterella* − IG *Akkermansia* − IG *Desulfovibrio* − IG *Intestinimonas* − IG	Untreated	In binge eating disorders, subjects had higher level of *Anaerostipes* and *Roseburia* and less *Sutterella*, *Akkermansia*, *Desulfovibrio* and *Intestinimonas.*
Morita et al. (2015)	Case Control	IG = 25 CG = 21	30.0 31.5	Diagnostic and Statistical Manual of Mental Disorders-IV-TR criteria (DSM-IV-TR)	Anorexia Nervosa	*Clostridium coccoides* − IG *Clostridium leptum* − IG *Bacteroides fragilis* − IG *Streptococcus* − IG *Lactobacillus* − IG	Untreated	In Anorexia Nervosa there was a decrease in *Clostridium coccoides, Clostridium leptum, Bacteroides fragilis*, *Streptococcus* and *Lactobacillus* taxa.
Borgo et al. (2017)	Case Control	IG = 15 CG = 15	25.6 24.4	Symptom Checklist-90 (SCL-90). Eating Disorder Inventory 2 (EDI-2). State Trait Anxiety Inventory (STAI). Beck Depression Inventory (BDI-II).	Anorexia Nervosa	*Pseudomonadota* + IG *Bacillota* − IG *Ruminococcaceae* − IG *Enterobacteriaceae* + IG *Ruminococcus* − IG *Roseburia* − IG *Clostridium* − IG	Daily food (filled in a three-day food record).	The composition of the intestinal microbiota was significantly affected by anorexia status at every taxonomic level. A negative correlation was detected only between BDI depression score and *Clostridium* genus.
Yuan et al. (2022)	Case Control	IG = 30 CG = 30	16.0 18.0	Diagnostic and Statistical Manual (DMS-V) and Hamilton Depression Scale (HAMD)	Anorexia Nervosa	*Lachnospiraceae* + IG *Enterobacteriaceae +* IG *Streptococcaceae* + IG *Coriobacteriaceae* + IG *Rikenellaceae* + IG *Ruminococcaceae* − IG *Bifidobacteriaceae* − IG *Peptostreptococcaceae* -IG *Oscillospiraceae* − IG *Burkholderiaceae* − IG	Untreated	AN patients showed a slight decrease in the richness and diversity. *Faecalibacterium and Synergistota* were significantly negative correlated with HAMD score.
Hemmings et al. (2017)	Case Control	IG = 18 CG = 12	42.0 38.7	Posttraumatic Stress Disorder Scale for DSM-5 (CAPS-5). Childhood Trauma Questionnaire (CTQ)	Posttraumatic stress disorder (PTSD).	*Bacillota* + IG CG *Bacteroidota* + IG CG *Pseudomonadota* + IG CG *Actinomycetota* − IG *Lentisphaerae* − IG *Verrucomicrobiota* − IG	Untreated	PTSD diagnosis was associated with decreased abundance of these phyla: *Actinomycetota*, *Lentisphaerae* and *Verrucomicrobiota*. *Actinomycetota* and *Verrucomicrobiota* were also associated with childhood trauma scores and Childhood Trauma Questionnaire (CTQ).
Schwarz et al. (2018)	Case Control	IG = 28 CG = 16	25.9 27.8	Hallucinations in the Brief Psychiatric Rating Scale — Extended (BPRS-E). The Scale for the Assessment of Negative Symptoms (SANS). Global Assessment of Functioning (GAF). Food habits were assessed by questions. Physical activity was assessed using the Gothenburg scale.	Psychotic episodes	*Lactobacillus* − IG *Lachnospiraceae* + IG *Ruminococcaceae* + IG *Bacteroides* + IG *Lactobacillaceae* + IG *Halothiobacillaceae* + IG *Brucellaceae* + IG *Micrococcineae* + IG Veillonellaceae − IG	Antipsychotics: Olanzapine, risperidone and quetiapine 20 days.	*Lactobacillus* taxon correlated positively with severity of psychotic symptoms and negatively with global assessment of functioning. *Lactobacillus*, *Lachnospiraceae*, *Ruminococcaceae* and *Bacteroides* spp. correlated negatively with global assessment of functioning.
Wang et al. (2020)	Case Control	IG = 26 CG = 24	4.3 4.5	The Autism Treatment Evaluation Checklist (ATEC) score prior to and following probiotics + FOS intervention or placebo Supplementation	Autism spectrum disorders (ASD)	*Rikenellaceae* + IG *Ruminococcus* + IG *Oscillospira* + IG *Odoribacter* + IG *Cetobacterium* + IG *Victivallales* + IG *Actinomycetota* − IG *Bifidobacteriaceae* − IG *Veillonellaceae* − IG *B. adolescentis* – IG (In comparison with the control group)	All participants received a Chinese-based diet provided by the hospital. Then children with ASD included in the second stage received probiotics + FOS or placebo. Intervention for 30–108 days.	Results showed that diversity of the gut microbiota in the ASD group was significantly different from that of the control group. *B. longum* was reduced in children with autism, other than *Clostridium* and *Ruminococcus,* which were increased in children with autism and probiotics +FOS intervention.
Kong et al. (2019)	Case Control	IG = 20 CG = 19	15.0 29.0	Patients had been diagnosed with ASD according to DSM-5 (Diagnostic and Statistical Manual of Mental Disorders) criteria. Lifestyle questionnaires.	Autism Spectrum Disorder (ASD)	*Bacillota* / *Bacteroidota* + IG *Pseudomonadota* + IG *Bacilli* + IG *Bacillota*/*Chloroflexi* + IG	Untreated	Significant *Pseudomonadota* overgrowth was associated with autism.
Wang et al. (2020)	Case Control	IG = 21 CG = 29	20.3 29.9	The trait anxiety was measured STAI, he Connor-Davidson Resilience Scale, The 15-item Mindful Attention Awareness Scale and Self-rating Depression Scale (SDS).	Anxiety	*Streptococcus* − IG *Blautia* − IG *Romboutsia* − IG *Escherichia, Shigella* − IG *Eubacterium hallii* group − IG *Eggerthella* − IG *Allorhizobium* − IG *Neorhizobium* − IG*Pararhizobium* − IG *Rhizobium* − IG	Mindfulness-based cognitive therapy (MBCT) 8 weeks	The intervention reported an increase in the abundance of the *Actinomycetota*, *Pseudomonadota, Fusobacterium*, *Streptococcus, Blautia, Romboutsia* and *Eggerthella* taxa.
Guo et al. (2022)	Case Control	IG = 44 CG = 30	35.3 40.2	Hamilton Anxiety Scale (HAMA-14), Five Factors Inventory-Neuroticism Subscale (FFI-N), The Chinese version of Illness Perception Questionnaire Revised (IPQ-R), The Twenty-Item Toronto Alexithymia Scale (TAS-20), Patient Health Questionnaire-15 (PHQ-15) and Cognitive Emotion Regulation Questionnaire-Chinese Version, CERQ.	Generalized Anxiety Disorder (GAD)	*Fusobacterium* − IG *Faecalibacterium* − IG *Meganomas* + IG	Untreated	F*usobacterium, Megamonas* and *Veillonella* were closely related to anxiety.
Tomova et al. (2015)	Case Control	IG = 10 Siblings = 9 CG = 10	2–9 5–17 2–11	The Childhood Autism Rating Scale (CARS) and Autism Diagnostic Interview (ADI).	Autism Spectrum Disorder (ASD)	*Bacillota* − IG *Bacteroidota* − IG *Clostridia* Cluster I + IG *Desulfovibrio* + IG	Dietary Supplementation of one capsule (*Lactobacillus, Bifidobacterium* and *Streptococcus*). Three times a day for 4 months.	After the probiotic implementation, the taxa *Bacillota, Bifidobacterium* and *Desulfovibrio* decreased, whereas *Bacteroidota* and *Lactobacillus* increased.

N, Sample size, IG, Intervention Group, CG, Control Group, + increase, − decrease.

**Table 2:** CASP quality assessment of the reviewed case–control papers.

	Section A: Are the Results of the Trial Valid?						Section B: What are the Results?			Section C: Will the Results Help Locally?		
Authors, Year	Item 1	Item 2	Item 3	Item 4	Item 5	Item 6	Item 7	Item 8	Item 9	Item 10	Item 11	Total Quality Score (0–11)
Kang et al. (2017)	Yes	Yes	Yes	Yes	Yes	No	Yes	Yes	Yes	No	Yes	9
Márquez-Morales et al. (2021)	Yes	Yes	Yes	Yes	Yes	No	Yes	Yes	Yes	No	Yes	9
Xu et al. (2017)	Yes	Yes	Yes	Yes	Yes	No	Yes	Yes	Yes	No	Yes	9
Leyrolle et al. (2021)	Yes	Yes	Yes	Yes	Yes	No	Yes	Yes	Yes	No	Yes	9
Morita et al. (2015)	Yes	Yes	Yes	Yes	Yes	No	Yes	Yes	Yes	No	Yes	9
Borgo et al. (2017)	Yes	Yes	Yes	Yes	Yes	No	Yes	Yes	Yes	No	Yes	9
Hemmings et al. (2017)	Yes	Yes	Yes	Yes	Yes	No	Yes	Yes	Yes	No	Yes	9
Schwarz et al. (2018)	Yes	Yes	Yes	Yes	Yes	No	Yes	Yes	Yes	No	Yes	9
Wang et al. (2020)	Yes	Yes	Yes	Yes	Yes	No	Yes	Yes	Yes	No	Yes	9
Kong et al. (2019)	Yes	Yes	Yes	Yes	Yes	No	Yes	Yes	Yes	No	Yes	9
Wang et al. (2020)	Yes	Yes	Yes	Yes	Yes	No	Yes	Yes	Yes	No	Yes	9
Chen et al. (2021)	Yes	Yes	Yes	Yes	Yes	No	Yes	Yes	Yes	No	Yes	9
Guo et al. (2022)	Yes	Yes	Yes	Yes	Yes	No	Yes	Yes	Yes	No	Yes	9
Yuan et al. (2022)	Yes	Yes	Yes	Yes	Yes	No	Yes	Yes	Yes	No	Yes	9
Tomova et al. (2015)	Yes	Yes	Yes	Yes	Yes	No	Yes	Yes	Yes	No	Yes	9

CASP, Critical Appraisal Skills Program. Item 1: Study issue clearly focused; Item 2: Cohort is recruited in an acceptable way; Item 3: Exposure is accurately measured; Item 4: Outcome is accurately measured; Item 5: Confounding factors are addressed; Item 6: Follow-up is long and complete; Item 7: Results are clear; Item 8: Results are precise; Item 9: Results are credible; Item 10: Results can be applied to the local population; Item 11: Results fit with available evidence.

**Table 3:** CASP quality assessment of the reviewed randomized controlled trial papers.

	Section A: Are the Results of the Trial Valid?						Section B: What are the Results?			Section C: Will the Results Help Locally?		
Authors, Year	Item 1	Item 2	Item 3	Item 4	Item 5	Item 6	Item 7	Item 8	Item 9	Item 10	Item 11	Total Quality Score (0–11)
Uemura et al. (2019)	Yes	Yes	Yes	No	Yes	Yes	Treatment improved statistically	Yes	No	Yes	Yes	9
Stevens et al. (2019)	Yes	Yes	Yes	Yes	Yes	Yes	Treatment improved statistically	Yes	No	Yes	Yes	10
Khine et al. (2020)	Yes	Yes	Yes	No	No	Yes	Treatment improved statistically	Yes	No	Yes	Yes	8

CASP, Critical Appraisal Skills Program. Item 1: Was the study issue is clearly focused?; Item 2: Was the assignment of patients to treatments randomized?; Item 3: Were all of the patients who entered the trial properly accounted for at its conclusion?; Item 4: Were patients, health workers and study personnel “blind” to treatment?; Item 5: Were the groups similar at the start of trial; Item 6: Aside from the experimental intervention, were the groups treated equally?; Item7: How large was the treatment effect?; Item 8:How precise was the estimate of the treatment effect?; Item 9: Can the results be applied to the local population, on in your context?; Item 10: Were all clinically important outcomes considered?; Item 11: Are the benefits worth the harms and costs?

### 3.1. Depressive symptoms and gut microbiota

After obese women followed a nutritional education program, microbial alpha diversity, and in particular *Bacteroidota* phylum, significantly decreased in the intervention group versus the control group. In contrast, there was a significant increase in several taxa of the *Bacillota* phylum, including *Lactobacillales*, *Streptococcus thermophilus* and *Veillonella parvula*. This increase was also observed in the *Actinomycetota* phylum, in particular the *Bifidobacterium* species (Uemura et al., 2019). However, in other studies, an increase in the *Bacteroidota, Verrucomicrobiota* and *Fusobacteriota* phyla was observed in a group with depression, while *Bacillota* was consistently enriched in the control group.

At genus level, an increase in taxa associated with neuroinflammation such as *Shigella* or *Escherichia* and others associated with HAMD scores such as *Anaerotruncus*, *Parabacteroides*, and *Anaeroglobus* were observed (Chen et al., 2021).

### 3.2. Attention deficit hyperactivity disorder (ADHD) and microbiota

Bacterial abundances and ADHD clinical ratings were not significantly associated. On the other hand, a significant correlation was observed between a higher relative abundance of *Bifidobacterium* and a lower ADHD-IV-RS score. No statistical differences were observed in the placebo group (Stevens et al., 2019).

### 3.3. Stress and gut microbiota

The composition of the gut microbiota in relation to stress showed significant differences in *Paraprevotella*, *Odoribacter*, *Veillonella*, and *Ruminococcus* genera, with reduced levels in chronic stressed endometriosis. In contrast, the prevalence of *Prevotella* was higher among chronic stressed patients than among healthy controls (Xu et al., 2017).

On the other hand, the effect of lactic acid bacteria-fermented beverage consumption on the gut microbiota of stressed students, specifically on three phyla: *Bacteroidota*, *Bacillota* and *Pseudomonadota*, revealed an increase in the *Bacteroidota* phylum. However, no significant differences were observed in the control group after the nutritional intervention in question. In the case of the *Bacillota* phylum, a significant increase was observed in both experimental and control groups. Finally, the *Pseudomonadota* phylum showed no significant differences between groups (Márquez-Morales et al., 2021).

### 3.4. Cognitive decline and gut microbiota

Cognitive function has been closely correlated with alterations in microbiota abundance. *Ruminococcus genus* positively correlated with all four cognitive functions (Recognition Trial, Memory Domain, Digit Span Backward and Semantic Fluency Span), *Coprococcus* correlated with Digit span backward, Color trails test 2 and Block Design, while *Parabacteroides* correlated with Digit span backward and Semantic fluency span. In contrast, the *Enterobacteriaceae* family was negatively associated with Block Design and Semantic Fluency Span, while the genus *Fusobacterium* was linked with Digit Span Backward and Color Trails Test 2 and the genus *Phascolarctobacterium* with Memory domain (Khine et al., 2020).

### 3.5. Binge eating disorder (BED) and gut microbiota

Differences in some bacterial genera have been observed in obese patients engaging in binge eating when compared to a control group. The BED subjects displayed very specific differences in their gut microbiota composition, exhibiting increased levels of *Anaerostipes* and *Roseburia* and a tendency toward elevated levels of *Bilophila* and *Bifidobacterium*. On the other hand, decreased levels of the bacterial genera *Sutterella*, *Akkermansia*, *Desulfovibrio* and *Intestinimonas* were observed in the same subjects (Leyrolle et al., 2021).

### 3.6. Anorexia nervosa and gut microbiota

Anorexia Nervosa status was characterized by an altered intestinal microbiota composition enriched in *Bacteroidota* and *Pseudomonadota* and depleted in *Bacillota*, *Ruminococcaceae*, *Ruminococcus* and *Roseburia*. This reduction in *Bacillota* was in line with the lower fecal butyrate concentration detected in the anorexia nervosa group (butyrate was negatively correlated with depression and anxiety) (Borgo et al., 2017). On the other hand, the *Enterobacteriaceae* family was more strongly represented than in control subjects (Borgo et al., 2017; Yuan et al., 2022). In addition, one study observed an interesting increase in the genus *Alistipes*, associated with depressive symptoms, and a reduction in *Faecalibacterium*, a great SCFA-producing taxon (Yuan et al., 2022). Moreover, fecal butyrate concentration and *Clostridium* were negatively correlated with anxiety and depression scores (Borgo et al., 2017). Furthermore, in a sample of Japanese women, a significantly decreased in *Clostridium coccoides* group, *Clostridium leptum, Bacteroides fragils* group, *Streptococcus* and *Lactobacillus* was observed in anorexia nervosa patients compared to the control group (Morita et al., 2015).

### 3.7. Posttraumatic stress disorder (PTSD) and gut microbiota

The phyla most represented in all participants (those with and without PTSD) were *Bacillota*, *Bacteroidota* and *Pseudomonadota*.

Moreover, in subjects with PSTD, the microbial communities correlated with clinical traits; there was a depleted abundance of two phyla (*Actinomycetota* and *Verrucomicrobiota*) that, in turn, correlated with a decrease in the CASP score. Furthermore, higher CTQ scores were also associated with lower abundance of *Actinomycetota* and *Verrucomicrobiota* (Hemmings et al., 2017).

### 3.8. Psychotic episodes and gut microbiota

The differences between cases and controls were not statistically significant. In contrast, the bacterial diversity among psychotic patients correlated with symptom severity. These correlations were observed for *Lachnospiraceae*, *Bacteroides* spp. and *Lactobacillus* taxa with respect to the total BPRS score. The positive symptoms were correlated with *Lactobacillus* and the negative symptoms were correlated with *Lachnospiraceae*, *Ruminococcaceae* and *Lactobacillus* (Schwarz et al., 2018).

### 3.9. Autism spectrum disorders (ASD) and gut microbiota

Bacterial alpha diversity at baseline was significantly lower in children with ASD than controls, though it increased following Microbiota Transfer Therapy. Specific genera were significantly altered after treatment, including *Bifidobacterium*, *Prevotella*, and *Desulfovibrio* genera. *Bifidobacterium* was underrepresented in children with ASD, in contrast to *Prevotella* and *Desulfovibrio*, which significantly increased after treatment. Initially, the relative abundance of *Prevotella* was similar in the control group and children with ASD. Thus, the data suggested that treatment resulted in several changes in the gut microbiota composition of ASD subjects compared to healthy controls (Kang et al., 2017). In a similar study, non-significant differences between autistic and neurotypical subjects were reported. The most abundant gut phyla in both ASD patients and control subjects were *Bacillota*, *Bacteroidota* and *Pseudomonadota*. In contrast, further analysis of dysbiosis markers revealed several differences in the gut microbiota composition of subjects with autism and their family member controls, including an increased ratio of *Bacillota*, *Bacteroidota*, *Pseudomonadota*, *Bacilli* and *Chloroflexi* taxa in the ASD group (Kong et al., 2019). According to Wang et al. (2020), neither the total bacterial community distribution nor the *Bacillota*/*Bacteroidota* ratio showed significant differences between ASD and control groups. In another study, Tomova et al. (2015) found statistically significant differences between the groups. In the ASD group there was a decrease in *Bacteroidota* phylum and an increase in *Lactobacillus*, *Clostridium* cluster I and *Desulfovibrio* compared to the control group, and the proportion between severe autism and mild autism was different. After probiotic supplementation with 3 strains (one *Bifidobacterium*, one *Streptococcus* and one *Lactobacillus*) in children with autism, the *Bacteroidota/Bacillota* ratio in their feces normalized, and the levels of *Bifidobacterium* and *Desulfovibrio* decreased. However, a significant decrease in the relative abundance of the *Actinomycetota* phylum was reported in the ASD group. In this regard, the structure of the gut microbiota in the ASD group differed significantly from that in the control group. There was a significant increase in the relative abundance of *Rikenellaceae*, *Ruminococcus*, *Oscillospira*, *Odoribacter*, and *Cetobacterium* taxa, and a significant reduction of *Actinomycetota*, *Veillonellaceae* and *Bifidobacterium*, particularly the *B. adolescentis* and *B. longum* species (Wang et al., 2020). According to the results of these different studies (Kong et al., 2019; Wang et al., 2020), *Bifidobacterium* levels are depleted in patients with ASD.

### 3.10. Generalized anxiety disorder (GAD) and gut microbiota

In Generalized Anxiety Disorder (GAD), an increase of *Fusobacterium* and *Megamonas* and a reduction of *Faecalibacterium* genus were observed compared to the control group. A positive and significant relationship was observed between the scores of the TAS, IPQ-R, PQH, FFI-N, HAMA and CERQ questionnaires with *Fusobacterium, Megamonas, Veillonella, Enterobacteriaceae* and *Bacteroidota*. On the other hand, the score of the PQH, FFI-N, HAMA and IPQ-R questionnaires was negatively and significantly associated with *Faecalibacterium* and *Ruminococcaceae* (Guo et al., 2022). In another study a reduction of *Actinomycetota* and an increase of *Bacillota taxa* were observed in subjects with anxiety. Moreover, the abundance of the genus *Subdoligranulum* was positively correlated with the trait anxiety scores (Wang et al., 2022).

## 4. Discussion

The present systematic review provides an update of studies characterizing the gut microbiota in psychiatric disorders and captures the large number of studies published in the recent years. We confirm an association between psychiatric disorders and the gut microbiota composition. The studies we reviewed demonstrate differences between subjects with psychiatric disorders and controls. In light of the reported results, the microbiota may be a regulator of mood disorders and behavior through the brain-gut-microbiota axis, thus positioning itself as a promising target in disease diagnosis and therapeutic interventions.

The studies included in the present review suggest that there is an alteration in microbial diversity in patients with depression compared to controls. Uemura et al. (2019) found that individuals with improved gut microbial diversity had a lower CES-D score, indicating better mental status, as shown by previous studies (Aizawa et al., 2016; Liang et al., 2018). Moreover, the results highlighted a correlation between gut microbiota and stress. In the case of endometriosis, a clear correlation has been demonstrated between the proinflammatory cytokines NF-ĸB p65 and COX2 and *Prevotella* genus (Hemmings et al., 2017; Xu et al., 2017; Márquez-Morales et al., 2021), and similar results have been reported for bacterial vaginosis caused by pathogenic and cytokine-producing *Prevotella* species such as *P. bivi* (Randis and Ratner, 2019). Therefore, exposure to stress would appear to change the composition of the intestinal microbiota in the body, and the microbiota in turn modulates stress levels (Foster et al., 2017; Ilchmann-Diounou and Menard, 2020). Nutritional intervention studies with a fermented drink containing *L. plantarum, L. paracasei* and *L. brevis* showed a beneficial effect on stress reduction, pointing to probiotics as a potential therapy in such cases (Márquez-Morales et al., 2021).

Khine et al. (2020) found significant differences in the gut microbiota between aging and healthy subjects. SCFA-producing and anti-inflammatory taxa have been correlated with improved brain function. In contrast, pro-inflammatory taxa, such as *Fusobacterium* or some members of the *Enterobacteriaceae* family, have been correlated with decreased brain function (Khine et al., 2020). Similarly, other studies have shown a relationship between microbiota and cognitive behavior. Over the aging period, the organism produces an inflammatory response that can affect the balance of the intestinal microbiota (Hu et al., 2016). Therefore, the intestinal microbiota in the elderly differs from that in healthy adults (Ni et al., 2019). The microbiota can influence complex behaviors, such as learning, stress, depression and anxiety (Cryan et al., 2019), leading us to conclude that the status of the brain may be influenced by inflammatory processes, which are strongly related with the brain-gut microbiota communication. With respect to eating disorders, few differences in bacterial genera have been observed between BED and non-BED groups, but the changes reported are of interest, namely, a reduction of bacteria that produce SCFAs. These bacteria play an important role in the regulation of inflammation, immunity and secretion of peptides related to ingestion behavior (Leyrolle et al., 2021). Moreover, *Akkermansia* depletion may affect the regulation of the intestinal barrier, increasing permeability and the risk of infection. In addition, the use of this bacterium in clinical trials, directly or as a postbiotic, could potentially be of use in weight loss and protection against diabetes (Depommier et al., 2019). Similar results have been observed in patients with PSTD, in whom a reduction of *A. muciniphila* was associated with higher host-mediated inflammation and increased intestinal permeability, suggesting a key role for this bacterium in the treatment of this pathology (Hemmings et al., 2017). In Anorexia Nervosa, an altered composition of the intestinal microbiota was observed, with a reduced proportion of carbohydrate-fermenting genera correlating with a lower butyrate concentration. Furthermore, a higher proportion of taxa (such as the *Enterobacteriacee* family) was associated with intestinal inflammation, which promotes bacterial translocation and systemic inflammation (Borgo et al., 2017), a tendency opposite to that seen in obese subjects (Rieder et al., 2017). These studies showed that proinflammatory cytokines are elevated in patients with eating disorders, so increasing SCFA in patients with anorexia nervosa, due to the production of taxa such as *Lachnospiraceae*, may help to improve host histone epigenetic states and decrease levels of inflammatory markers (Morita et al., 2015; Yuan et al., 2022). Therefore, gut microbiota composition may affect the total amount of energy extracted from food intake, which is relevant for weight regulation (Seitz et al., 2020). The effect of the gut microbiota on patients with first-episode psychosis (FEP) is also relevant. Schwarz et al., 2018 identified differences in the gut microbial composition between FEP-patients and controls in relation to the severity of psychotic symptoms and global functioning assessment. A decrease in butyrate-producing taxa such as *Faecalibacterium*, *Blautia*, *Ruminococcus* or *Roseburia* could be a factor influencing the severity of symptoms (Schwarz et al., 2018). Similarly, in different studies on neurodevelopmental diseases (Kang et al., 2013; Vuong and Hsiao, 2017), the gut microbiota dysbiosis was identified in ASD subjects, who exhibited a higher abundance of pathogenic bacteria and a lower number of beneficial taxa, reduced levels of SCFAs and several metabolic disorders, in this sense a strong correlation was observed in *Desulfovibrio* with the severity of autism manifestations. Following an intervention consisting of a prebiotic compound and several probiotics, such as *B. longum* and *Lactobacillus* (*L. paracasei* and *L. rhamnosus*), a change in the gut microbiota was observed, as it became more similar to that of control children (Tomova et al., 2015), in addition to an increase in fermenting taxa that contributed to a significant increase in the concentration of SCFAs and an improvement in the metabolism of dopamine and tryptophan. In fact, microbiota modulation was shown to lead to an improvement in the health of ASD patients (Wang et al., 2020). Similar results showed that the gut microbiota is related to gastrointestinal symptoms and ASD (Kang et al., 2017; Kong et al., 2019). In the light of all this research, understanding the role of the microbiota in brain development should be a therapeutic target of future research into mental illness (Gárate et al., 2011; Sandhu et al., 2017; Valles-Colomer et al., 2019), since multiple studies have demonstrated this relationship (Hu et al., 2016; Borgo et al., 2017; Colica et al., 2017) and its influence on the psychoneuroimmunology network (El Aidy et al., 2015). More robust studies should be performed to demonstrate causality.

There are several limitations to the studies reviewed herein. It is very difficult to prove causality between bacterial taxa and these diseases due to the lack of both sufficient statistical power and consensus concerning interpretations of microbiota data. In many of the studies analyzed the sample size was too small to draw conclusions. In addition, there were many differences found in specific microbial members, although this could be linked to issues with the methodology and not with real inconsistency between groups. The absence of a metagenomic study of the patient’s intestinal populations over time preclude from performing a causal correlation. There are differences at the metagenomic sequencing level with some studies showing low sequences per sample that limit the results and should be taken into account when making comparisons between studies. Exogenous sources of inter-study heterogeneity (antidepressants, diet, sex) were also observed, such as Morita et al. (2015), that did not take into account the psychiatric medication of the subjects, if they were prescribed, or Guo et al. (2022), that took into account psychoactive substances, but not psychiatric medication. In addition, one of the most prominent limitations that can be observed is the inconsistency found in the different methods used for recruitment, screening and categorization of cases and controls, as many diagnoses are carried out by a self-reported methodology. In this sense, Wang et al. (2020) sampled medical subjects that represent a possible bias. In relation to age, Yuan et al. (2022) recruited both children and adults for the sample, which is quite heterogeneous, whereas Tomova et al. (2015) included two-year-old children with autism in the study, with the diagnosis being premature at such age (Ghosn et al., 2022). Another limitation is that all psychiatric symptoms in the reviewed studies should match the DSM-5 diagnostic criteria. Furthermore, in most studies the recruitment is at a local level so the diversity and composition of the gut microbiota of that population may not be representative of that from other countries or continents, making comparison more problematic. Further studies are needed to determine whether gut microbiota dysbiosis is a cause or an effect of the disease. In addition, given the heterogeneity of the studies, meta-analysis cannot be performed.

## 5. Conclusion

The articles evaluated for this review show a clear dysbiosis of the gut microbiota in all the psychiatric disorders studied: depression, stress, brain function, eating disorders, psychotic episodes and ASD. In general, a reduction of fermenting taxa has been observed, parallel to a decrease in SCFAs concentrations. This occurs in addition to an increase in certain opportunistic or pathogenic bacteria that can maintain a proinflammatory status. Interventional studies with prebiotic fibers, probiotics (especially *Lactobacillus* and *Bifidobacterium* species) or postbiotics (for example, pasteurized compounds of *A. muciniphila*) have shown the possibility of mitigating the symptoms associated with these diseases. Thus, it is feasible that the status of the brain is influenced by inflammatory processes, which reinforces the theory of a gut-brain axis communication.

## Data availability statement

The original contributions presented in the study are included in the article/Supplementary material, further inquiries can be directed to the corresponding author.

## Author contributions

CG-DV and JF: conceptualization, formal analysis, investigation, methodology, validation, visualization, writing—original draft, and writing—review and editing. ES, IM-C, and CM: supervision, validation, visualization, and writing—review and editing. CB: conceptualization, formal analysis, funding acquisition, supervision, validation, visualization, and writing—review and editing. All authors contributed to the article and approved the submitted version.

## Funding

This study was funded by a grant (PI18/00932 and PI21/01160) from the Institute of Health Carlos III and the European Regional Development Fund (ERDF, “A Way of Doing Europe”), CIPROM/2022/32 from Generalitat Valenciana and UGP-20-132 from FISABIO. CG-DV was a beneficiary of a PFIS contract (FI19/00076), JF was a beneficiary of a Plan de Ciencia, Tecnología e Innovación 2018-2022 del Principado de Asturias contract from Ficyt (Principado de Asturias, AYUD/2021/58584), and CB was a beneficiary of a Miguel Servet type I contract from the Institute of Health Carlos III (CP19/00077).

## Conflict of interest

The authors declare that the research was conducted in the absence of any commercial or financial relationships that could be construed as a potential conflict of interest.

## Publisher’s note

All claims expressed in this article are solely those of the authors and do not necessarily represent those of their affiliated organizations, or those of the publisher, the editors and the reviewers. Any product that may be evaluated in this article, or claim that may be made by its manufacturer, is not guaranteed or endorsed by the publisher.
